# Lymphoid Aggregates That Resemble Tertiary Lymphoid Organs Define a Specific Pathological Subset in Metal-on-Metal Hip Replacements

**DOI:** 10.1371/journal.pone.0063470

**Published:** 2013-05-28

**Authors:** Saloni Mittal, Matthew Revell, Francesca Barone, Debbie L. Hardie, Gulraj S. Matharu, Alison J. Davenport, Richard A. Martin, Melissa Grant, Frederick Mosselmans, Paul Pynsent, Vaiyapuri P. Sumathi, Owen Addison, Peter A. Revell, Christopher D. Buckley

**Affiliations:** 1 Rheumatology Research Group, Institute of Biomedical Research, MRC Centre for Immune Regulation, University of Birmingham, Birmingham, United Kingdom; 2 Royal Orthopedic Hospital, Birmingham, United Kingdom; 3 Biomaterials Unit, School of Dentistry, University of Birmingham, Birmingham, United Kingdom; 4 School of Metallurgy and Materials, University of Birmingham, Birmingham, United Kingdom; 5 School of Engineering and Applied Sciences & Aston Research Centre for Healthy Ageing, University of Aston, Birmingham, United Kingdom; 6 Diamond Light Source, Harwell Campus, Didcot, United Kingdom; University of California San Francisco, United States of America

## Abstract

Aseptic lymphocyte-dominated vasculitis-associated lesion (ALVAL) has been used to describe the histological lesion associated with metal-on-metal (M-M) bearings. We tested the hypothesis that the lymphoid aggregates, associated with ALVAL lesions resemble tertiary lymphoid organs (TLOs). Histopathological changes were examined in the periprosthetic tissue of 62 M-M hip replacements requiring revision surgery, with particular emphasis on the characteristics and pattern of the lymphocytic infiltrate. Immunofluorescence and immunohistochemistry were used to study the classical features of TLOs in cases where large organized lymphoid follicles were present. Synchrotron X-ray fluorescence (XRF) measurements were undertaken to detect localisation of implant derived ions/particles within the samples. Based on type of lymphocytic infiltrates, three different categories were recognised; diffuse aggregates (51%), T cell aggregates (20%), and organised lymphoid aggregates (29%). Further investigation of tissues with organised lymphoid aggregates showed that these tissues recapitulate many of the features of TLOs with T cells and B cells organised into discrete areas, the presence of follicular dendritic cells, acquisition of high endothelial venule like phenotype by blood vessels, expression of lymphoid chemokines and the presence of plasma cells. Co-localisation of implant-derived metals with lymphoid aggregates was observed. These findings suggest that in addition to the well described general foreign body reaction mediated by macrophages and a T cell mediated type IV hypersensitivity response, an under-recognized immunological reaction to metal wear debris involving B cells and the formation of tertiary lymphoid organs occurs in a distinct subset of patients with M-M implants.

## Introduction

Total hip replacement is the most successful surgical procedure for the long-term alleviation of pain and disability in patients with hip arthritis [1]. The cellular reaction to wear particles in the tissue around total joint replacements has long been considered to be a significant contributor to aseptic implant loosening [2]. Metal-on-polyethylene (M-P) bearing surfaces have historically been the standard articulation. However these bearings predominantly generate polyethylene wear debris, the cellular reaction to which gives rise to local bone loss [2]. High-carbon, cobalt-chromium-molybdenum alloys have returned to prominence with hip resurfacing surgery and have increasingly been used over the last decade [3], [4], [5].

Recently, there has been concern expressed over the biological responses to metal wear debris occurring in some individuals with Metal-on-metal (M-M) hips. A number of terms have been used in the literature to describe both the clinical and pathological entities associated with M-M bearings [2], [6], [7], [8], [9], [10]. However, there is no clear consensus defining the boundaries of each of these terms [11]. Adverse reaction to metal debris (ARMD) is an umbrella term that encompasses a spectrum of findings and includes metallosis, pseudotumour, ALVAL (aseptic lymphocyte-dominated vasculitis-associated lesion), and macroscopic tissue necrosis [9]. Metallosis is the macroscopic staining of the soft-tissues and is associated with abnormal wear [11]. Pseudotumours are non-neoplastic, non-infective, solid or semi-liquid soft-tissue periprosthetic masses associated with M-M hip bearings which can progress to soft-tissue destruction with subsequent poor clinical outcomes [8], [12], [13], [14]. The entity ALVAL was introduced to describe a specific adverse histological reaction to metal wear debris [7]. ALVAL is characterised by tissue necrosis, fibrin, perivascular lymphocytes, lymphoid aggregates containing B and T cells, and plasma cells [7].

While it has been recognised that sensitisation occurs in some individuals receiving metal implants, with a T cell mediated type IV hypersensitivity response occurring in the local soft tissues [15], controversy exists regarding whether this hypersensitivity is indeed the dominant biological reaction responsible [16]. In addition, it is not yet understood how the recently-described presence of both B and T cells in the periprosthetic tissues of failed M-M bearings fits with the clinical and histopathological picture [7], [8], [14], [15], [17], [18], [19], [20], [21], [22].

The B and T cell-containing aggregates seen in ALVAL, bear a remarkable resemblance to tertiary lymphoid organs (TLOs) described in tissues of several chronic inflammatory diseases [23] such as the joints of rheumatoid arthritis (RA) [24], [25], [26], the salivary glands of Sjogren's syndrome [27], [28], and the thyroid of Hashimoto's thyroiditis [29]. The clinical relevance of these structures is debated with evidence that associates lymphoid follicles with more aggressive disease and a worse clinical outcome [26], [30]. Functionality of TLOs has been recognized in terms of local expression of activation-induced cytidinedeaminase (AID), the enzyme responsible for class-switch recombination (CSR) and somatic hypermutation (SHM) and differentiation of plasma cells, establishing the critical link between TLO formation and humoral immunity [31], [32], [33]. The question therefore arises as to whether the B and T cell aggregates found in periprosthetic tissues of M-M hip arthroplasties are in fact lymphoid follicles of the type recognised as TLO's and whether this histological presentation is associated with a defined disease process and clinical prognosis. Here we provide the first description of ALVAL as TLO, by investigating the cellular and molecular features typical of tertiary lymphoid organs in these specific periprosthetic tissues.

## Materials and Methods

### Review of clinical material

Local ethics committee approval was obtained for this study from North West 5 Centre for Research Ethics Committee (REC Reference 09/H1010/75) and the NRES Committee South West, Bristol REC centre (REC Reference 12/SW/0088). All patients gave written consent and the ethics committees approved the consent procedure. Sixty-two cases of M-M hip resurfacings and total hip replacements undergoing revision surgery at a single specialist arthroplasty centre between 1998 and 2012 were included. Cases were identified from the clinical and histopathology databases of the hospital. All revisions were performed for suspected adverse reactions to metal debris. Cases were excluded if there was microbiological or histological evidence of infection, or if the index procedure was performed for rheumatoid arthritis. The archival paraffin embedded sections containing periprosthetic tissue taken at the time of revision surgery were anonymised by the allocation of a study number. Two histopathologists (P.A.R and V.P.S), blinded to the clinical details and the cellular pathology diagnostic reports, reviewed all sections.

### Characterisation of histopathological appearances

Five µm formalin fixed paraffin embedded tissue sections underwent routine staining with haematoxylin-eosin (H&E) to determine the presence of individual histological features found in relation to failed prosthetic joints (2). Lymphocytic and macrophage infiltrates were assessed and on the basis of their distribution classed as either diffuse, perivascular or focal aggregates. Further characterization of the aggregates was achieved by staining sequential sections with pan T (CD3) and pan B (CD20) cell markers by immunohistochemistry. Briefly, sections were deparaffinised and rehydrated through xylene and graded ethanol solutions. After washing in running water, antigen retrieval was carried out by incubating the sections in epitope retrieval solution at pH 8 (Novocastra RE7116) for 16 hours at 68°C. Following incubation with primary antibody for 1 hour at room temperature at optimised dilutions (CD3, 1:50; CD20, 1:600), the brown colour reaction with diaminobenzidine (DAB) was developed using ChemMateEnVision detection kit (Dako) according to the manufacturer's instructions. Tonsil and reactive lymph node were used as positive controls.

### Characterization of tertiary lymphoid tissue

For immunofluorescence and double immunohistochemistry, formalin fixed paraffin embedded 5 µm tissue sections were treated with W-CAP TEC buffer, pH 8.0 (Bio Optica, Milan, Italy) for 40 min at 98°C for deparaffinisation and antigen retrieval.

### Immunofluorescence

For triple staining with immunofluorescence, the sections were washed and blocked with 10% FCS in PBS for 30 min and incubated with primary antibodies overnight at 4°C. To avoid cross reactivity, primary antibodies from different species or different mouse sub-classes were used in combination. After three washes in PBS, the sections were incubated with the appropriate secondary antibodies for one hour at room temperature. The secondary antibody cocktail also contained Hoechst dye for nuclear staining. After the final three washes with PBS, slides were mounted in 2.4% w/v 1,4-diazabicyclo (2,2,2) octane (Aldrich, Gillingham, England) in 90% v/v glycerol (Fisons Scientific, Loughborough, UK) in PBS (pH, 8.6) and analysed by confocal microscopy (LSM 510; Carl Zeiss Ltd, Cambridge, England).

### Immunohistochemistry

Single or double antigen labelling immunohistochemistry (IHC) was performed to visualise CXCL13 and CCL21. Briefly, after dewaxing and antigen retrieval, the sections were incubated with BLOXALL (Vector laboratories) for 10 min to block any endogenous peroxidase and alkaline phosphatase activity. After washing with TBS pH 7.6, the sections were incubated with primary antibodies for 1 hour at room temperature and then washed. Sections were incubated for 45 min at room temperature with either donkey anti-sheep IgG biotinylated antibody and anti-mouse IgG-HRP (Mouse ImmPRESS kit, Vector Laboratories), or with donkey anti-sheep IgG biotinylated antibody and anti-rabbit IgG-HRP (Rabbit ImmPRESS kit, Vector Laboratories). This was followed by incubation with a preformed avidin and biotinylated alkaline phosphatase macromolecular complex (VECTASTAIN^®^ ABC-AP kit, Vector Laboratories). After the final wash in TBS pH 7.6 brown colour was developed using ImmPACT DAB Peroxidase Substrate (Vector laboratories) and subsequently red colour was developed using vector red alkaline phosphatase substrate kit (Vector laboratories). Finally the sections were counterstained with haematoxylin and mounted in glycergel (Dako, Cambridge). Images were acquired using Leica DM6000 photomicroscope (Leica Microsystems, UK) and analysed with QCapture Pro software.

### Antibodies

The primary and secondary reagents used for immunofluorescence and immunohistochemistry are: *Primary antibodies*: CD3 (polyclonal rabbit) Dako; CD3 (clone PS1) Novocastra; CD20 (clone L-26) Dako; GP38 (clone D2-40) Serotec; CD138 (clone B-A38) Serotec; CD21 (clone 2G9) Dako; PNAd (clone MECA-79) BD Pharmigen; CXCL13 (polyclonal goat AF801) R&D systems; CCL21 (polyclonal goat AF366) R&D systems; Baff (polyclonal rabbit) Chemicon; April (clone April-2) Enzo life sciences. *Secondary antibodies*: Anti-mouse IgG1 FITC, Southern Biotechnologies; Anti-fluorescein Alexa 488, Invitrogen; Anti-mouse IgG2b TRITC, Southern Biotechnologies; Anti-rat IgM Alexa 649, Invitrogen; Anti-rabbit IgG Cy5, Jackson ImmunoResearch; Anti-rabbit IgG TRITC, Jackson ImmunoResearch; Anti-rabbit IgG TRITC, Jackson ImmunoResearch; Anti-sheep biotin, The Binding Site.

### Confocal microscopy

Images were acquired on LSM 510-UV confocal microscope and analysed by the LSM510 Image Examiner software. For quantification of T and B cells the relative percentage of CD3 and CD20 cells were determined by counting the number of positive pixels/area of aggregate for both CD3 and CD20 channels.

### Statistical analysis

Graphpad prism5 was used for quantification and statistical analysis, and the Mann-Whitney test was performed to determine differences between subgroups. A p-value of <0.05 was considered to be statistically significant.

### Synchrotron X-ray fluorescence measurements

Frozen sections (4–6 µm thickness) were cut using a cryotome from tissue that had been snap frozen in liquid nitrogen after surgical retrieval in order to maintain the distribution and speciation of implant-derived metallic elements. A tungsten carbide knife was used to prevent spurious contamination with cobalt (Co) and chromium (Cr). Preliminary histological investigations of the tissue block confirmed the presence of TLO-like structures. Alternating sections were reserved for haematoxylin and eosin staining; elemental mapping using synchrotron X-ray fluorescence (XRF) or immunofluorescence. Sections for synchrotron XRF measurements were mounted on ultra-pure fused silica plates possessing trace concentrations of Co and Cr at <10 ppb (Spectrosil 2000, Heraeus Quarzglas GmbH & Co., Hanau, Germany). To comply with experimental facility protocols for unfixed human tissue, the sections were covered with a 4 µm Ultralene film (SPEXSamplePrep., NJ, USA) which was secured at the periphery of the glass slide with epoxy resin. Synchrotron micro-focus XRF measurements were undertaken using a 4-element Si drifts detector on the I18-Beamline at the Diamond Light Source (Oxfordshire, UK) using an incident energy of 10 keV. Tissue sections were initially mapped using a 50 µm resolution to identify areas of interest. Subsequently, the incident beam was focused to give a spot size 5 µm (height) by 3.4 µm (width) and the sample was mounted at a 45° angle to the incident beam, thus resulting in a beam footprint of 5×5 µm and providing a resolution at a length scale similar to that of individual cells. Data was calibrated using the incident, Ar and Fe energy peaks prior to background correction and peak fitting for Co and Cr using PyMCA software [34]. Elemental maps for Co and Cr of regions consistent in appearance with TLO structures were correlated with the underlying histology and immunohistochemistry using the adjacent sections stained either with H&E or for immunofluorescence as references. XRF maps were overlaid on confocal microscopy images enabling statistical comparison of the fitted Co and Cr fluorescence values between regions with T and B cell infiltrates; with disorganised immune cell infiltrates and with the acellular background levels. One way analyses of variance (ANOVA) and post-hoc Tukey tests were performed at a 95% significance level.

## Results

### Histopathological review of periprosthetic tissues from revised M-M hips

Histological examination of 62 periprosthetic tissue samples from M-M hip bearings demonstrated macrophages with variable amounts of intracellular metal wear debris in 55 cases (89%), with the macrophages present in the remaining 7 cases (11%) containing no apparent particles. Lymphocyte populations were present in 51 cases (82%) with 11 samples (18%) lacking any significant lymphocytic infiltrate.

**Figure 1:**
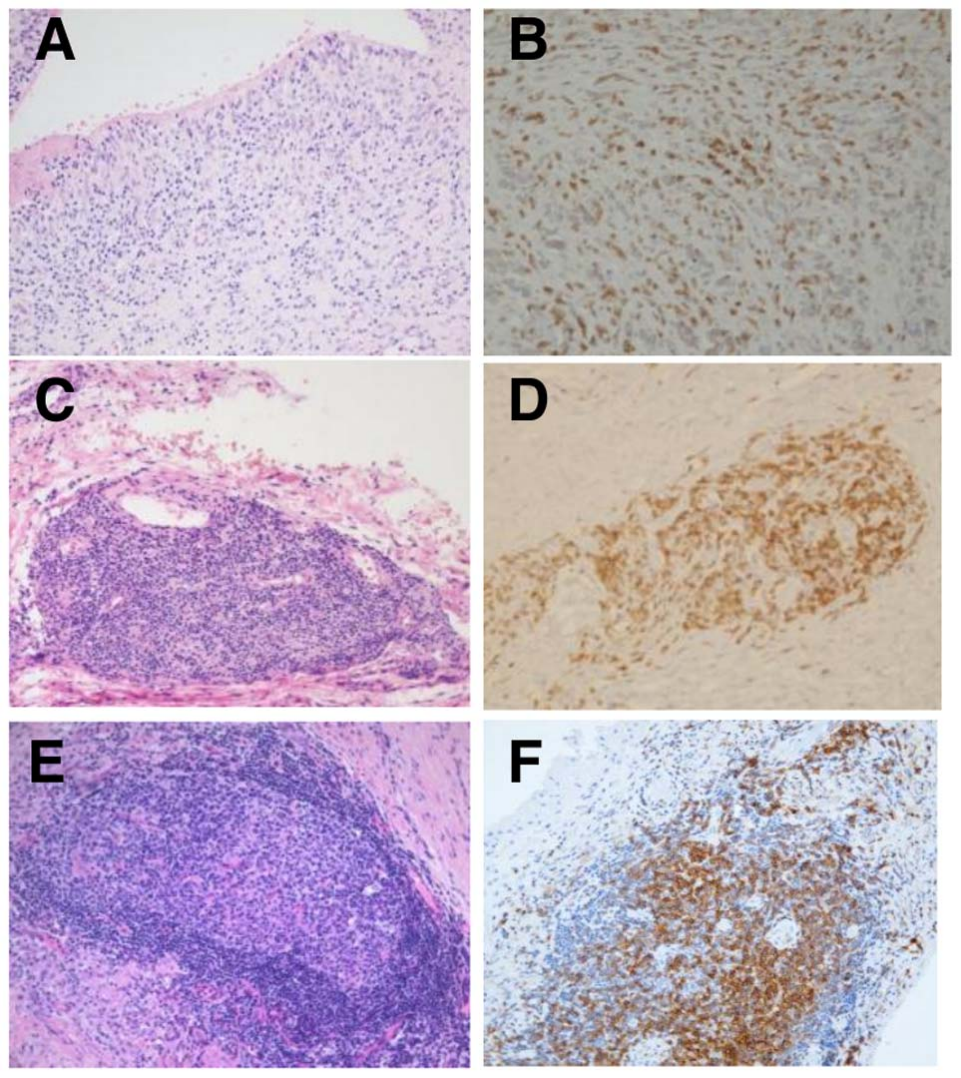
Histopathological review of periprosthetic tissues from hip joint M-M revision surgery. Histopathological appearances in formalin fixed sections showing group 1: diffuse lymphocyte infiltrate (A), group 2: T cell aggregate (C), and group 3: organised lymphoid aggregate with central follicular area (E). Immunohistochemistry with CD3 shows diffuse (B), T cells in an aggregate (D) and T cells mixed with B cells (not stained) in a follicular type of aggregate (F). Images taken at 10X magnification.

The distribution of lymphocyte populations determined from H&E sections and immunohistochemistry enabled subdivision of the 51 cases with lymphocytic infiltrate into three distinct groups, namely: a) diffuse lymphocyte infiltration with no aggregates (group 1); b) lymphocyte aggregates containing predominately T cells (group 2); c) lymphocyte aggregates containing T and B cells (group 3). A diffuse T cell infiltrate was seen in 26 cases (51%) in which there were no aggregates (Figure 1A, B), T cell aggregates were seen in 10 cases (20%) (Figure 1C, D) and organized T and B cell aggregates, with appearances of TLOs were present in 15 cases (29%) (Figure 1E, F).

### T/B cell segregation, formation of FDC networks and High Endothelial Venules (HEVs)

**Figure 2:**
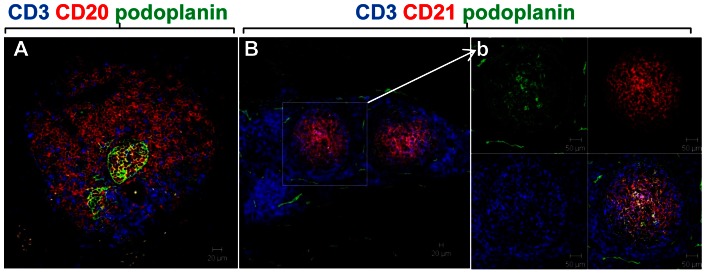
Lymphoid follicles resembling ectopic tertiary lymphoid organs occurs in a distinct subset of patients with lymphoid-like aggregates. A) Immunofluorescence (IF) from tissues of M-M implants with lymphoid follicles show central B cell rich area (red) surrounded by T cells (blue) and reticular pattern of podoplanin expression in the B cell area (green). (B, b). IF images showing co-localisation of CD21+ FDC network (red) with podoplanin (green). FDC network stained with CD21 and podoplanin are largely excluded from T cell area (blue).

Periprosthetic tissues from all 15 patients belonging to group 3 were further characterised for hallmark features of tertiary lymphoid tissue including segregation of T cells and B cells into discrete areas, presence of follicular dendritic cells (FDCs) indicative of a germinal centre-like reaction and acquisition of high endothelial venule (HEV)-like phenotype by blood vessels. In order to assess the compartmentalisation of T and B cells into discrete areas along with the formation of FDC network, sequential sections were stained by triple immunofluorescence using markers for T cells (anti-CD3), B cells (anti-CD20), and the lymphoid stromal cell marker, podoplanin (Figure 2). All 15 samples showed aggregates of CD3+ T cells distributed loosely surrounding centrally placed CD20+ B cells (Figure 2A). In a large percentage of the foci (55%), a histologically recognisable germinal centre could be identified in the B cell-rich zone. Intense podoplanin staining was consistently detected in this area assuming the typical reticular pattern usually associated with an FDC network (Figure 2A, B) as well as on lymphatic vessels outside the germinal center. FDC identity was further confirmed by double immunofluorescence using antibody to podoplanin as well as CD21 (marker for FDCs) that showed co-localization between the two markers (Figure 2b).

Quantification of B and T cells, the percentage of segregation in T/B cell discrete areas and the presence of FDC networks were performed on a total of 65 lymphoid aggregates present in the 15 samples. On average, the inflammatory foci contained a larger percentage of T cells (60%) as compared to B cells (40%) with scarce variability among aggregates from the same or different samples (P<0.001). Only 40% of all the lymphoid aggregates were found to be segregated in discrete T/B cell areas. No correlation was found between the percentage of B cells infiltrating the samples and the degree of T/B cell segregation. As expected, FDCs were detected in a high percentage (range 60-70%) of segregated aggregates.

**Figure 3:**
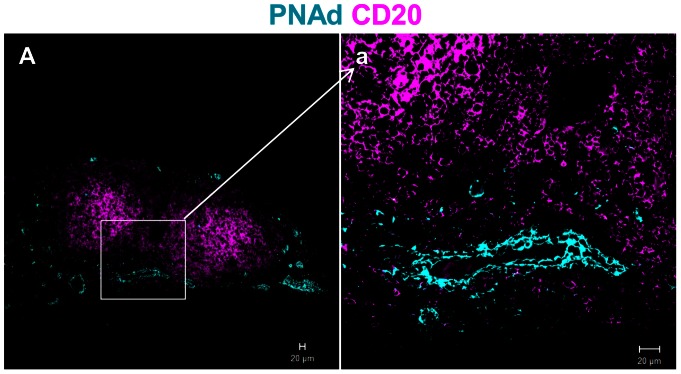
Presence of HEVs in ectopic lymphoid follicles. Paraffin sections of periprosthetic tissues having large lymphoid-like aggregates were stained with marker for B cell, CD20 (pink) and marker for HEVs, PNAd (cyan). Shown are representative images from the group containing large lymphoid aggregates. Plump HEVs expressing PNAd were detected in the periphery of lymphoid aggregates, in the T cell area (A, inset a).

The activated blood endothelial cells of secondary and tertiary lymphoid organs acquire a HEV-like phenotype by expressing adhesion molecules and the peripheral node adresin (PNAd), which aids the recruitment of circulating T and B cells into these tissues [35]. Using antibody to PNAd (MECA-79), the presence of HEVs was detected in the tissues of all 15 patients from group 3. HEVs were found in the T cell areas, at the periphery of the aggregates (Figure 3) or in the inter-follicular area. PNAd+ HEVs were detected in all samples containing large organised follicles.

### Expression of the constitutive chemokines CXCL13 and CCL21

**Figure 4:**
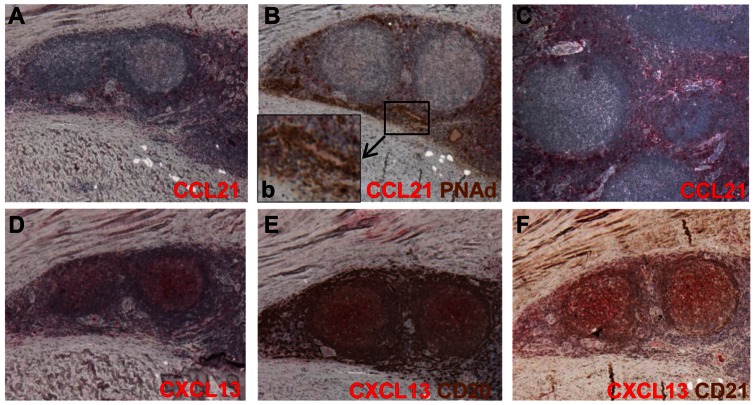
Expression of the chemokine CCL21 and CXCL13 in ectopic lymphoid follicles. Photomicrographs of 5 µm thick sections with immunohistochemical staining of CCL21 and CXCL13 show scarce expression of CCL21 outside the germinal centre, in the T cell area (red in A) and intense CXCL13 staining in the B cell rich areas (red in D). Double immunohistochemistry show minimal expression of CCL21 (red in B, inset b) with PNAd+ HEVs (brown in B, inset b). Positive CCL21 expression is detected in control tonsil (red in C). CXCL13 expression (red in E, F) is detected in the CD20+ B cell-rich areas (brown in E) and CD21+ follicular dendritic cell network (brown in F). Images taken at 10× magnification.

The expression of the lymphoid homing chemokines, CCL21 and CXCL13, has been shown to be critical for recruitment of T and B cells and their compartmentalisation into functional zones during development of lymphoid organs and in tertiary lymphoid organ formation [24], [27], [28], [36], [37]. The expression of the chemokines CCL21 and CXCL13 in organised lymphoid aggregates was studied by single immunohistochemistry (Figure 4A, D). Double immunohistochemistry was also used to study the expression of CXCL13 in association with either CD20+ B cells or the CD21+ FDC network and CCL21 expression in association with PNAd+ HEVs (Figure 4B, b, E, F). CXCL13 expression was mainly detected in the B cell rich area and in association with the FDC network within the germinal centre like structures (Figure 4E, F). Scarce expression of CCL21 could be detected in the T cell areas of large highly organised lymphoid aggregates (Figure 4A). Little CCL21 expression was observed in association with PNAd+ HEVs (Figure 4B, inset b), unlike that classically observed in secondary lymphoid organs. Positive CCL21 staining was demonstrated on tonsil (Figure 4C).

### Expression of B cell survival and proliferation factors, Baff and April

**Figure 5:**
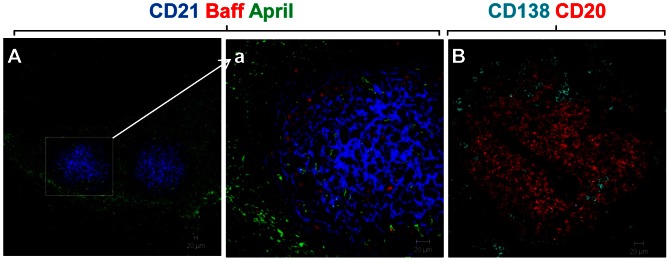
Expression of the B cell survival factors BAFF and April in ectopic lymphoid follicles and the presence of plasma cells in ectopic lymphoid follicles. Immunofluorescence images of staining of BAFF (red) and April (green) detected in the CD21+ follicular dendritic cell network (blue) (A) Paraffin sections of tissues showing organised lymphoid aggregates were also stained with a marker for plasma cells, CD138 (cyan), and B cells (CD20) (red) by double immunofluorescence (B). Abundant plasma cells were detected at the periphery of the aggregates as well as in the surrounding tissue. Shown are representative images from the group of cases containing organised lymphoid aggregates.

Baff (B cell activating factor) and April (A proliferation inducing ligand) are members of the TNF superfamily that have been shown to play a crucial role in survival, activation and proliferation of B cells as well as the maintenance of the plasma cell compartment in secondary lymphoid organs [38], [39]. Since the lymphoid aggregates of group 3 showed a high percentage of B cells (as compared to the other groups), we studied the expression of Baff and April in these tissues by immunofluorescence. Expression of Baff was mainly detected within the germinal centre in association with FDC networks. April expression showed a diffuse distribution within the tissue with greater intensity observed inside the lymphoid aggregates (Figure 5A, inset a). Abundant plasma cells labelled positively for syndecan (CD138) were confirmed as present either at the periphery of the aggregates or dispersed within the surrounding tissue (Figure 5B), suggesting a functional role of the foci in the process of B cell affinity maturation and local plasma cell differentiation.

### Co-localisation of implant derived metal ions with TLO structures

**Figure 6:**
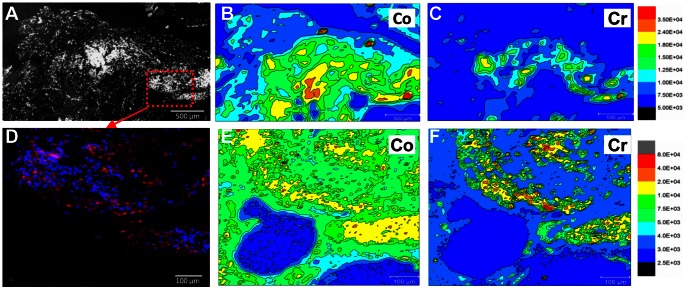
Co-localisation of implant derived metallic elements, Co and Cr, with TLO structures. Confocal nuclear density map (A) and overlaid X-ray fluorescence maps for Co (B) and Cr (C) measured at a 50 μm resolution demonstrate a strong co-localisation of Cr with the B and T cell infiltrate. Scales refer to fluorescence intensity in arbitrary units. (D) is an immunofluorescence image taken from the boxed region in (A) showing B cells (red) and T cells (blue). Micro-focus X-ray fluorescence maps of the same region (5 μm resolution) demonstrates Co to be more evenly dispersed (E) with Cr well co-localised with the immune cell infiltrate (F).

In order to explore whether the formation of TLO's was associated with the presence of implant metal derivatives in synovial tissue, we performed synchrotron XRF measurements for cobalt and chromium in synovial tissue samples from M-M hip replacements where lymphoid aggregates occurred. Micro-focus XRF measurements at a 5 µm resolution allowed visualisation of Co and Cr distributions at a similar length-scale to individual cells. Superimposition of synchrotron XRF measurements with the underlying cellular content demonstrated a clear co-localisation (significantly increased regional concentrations) of the principle M-M hip implant metallic elements, Co and Cr, with regions of dense T and B cell infiltration (Figure 6A–E). One-way ANOVAs and post-hoc Tukey tests demonstrated that the mean Co and Cr fluorescence values were significantly increased in all areas of the synovial tissue when compared with background levels (P<0.001). However, both Co and Cr were encountered in significantly elevated levels in regions of T and B cell infiltration when compared to non-infiltrated regions of the tissue (P<0.001). Cr in particular appeared concentrated in such TLO regions demonstrating a 2.9 fold increase in fluorescence values compared with a 2.0 fold increase in the Co signal.

## Discussion

Osteolysis and aseptic loosening associated with traditional M-P implants is mainly considered to be driven by a cell-mediated reaction, involving a general foreign body reaction by macrophages, multinucleate giant cells and T lymphocytes [2]. In contrast, the presence of lymphoid aggregates containing T and B cells, plasma cells, and a perivascular lymphocytic infiltrate, interpreted by some as a vasculitis, are considered to be unique features of ALVAL associated with M-M bearings [7], [9], [10], [14], [40]. In this study, we reviewed and classified the histopathological changes in periprosthetic tissues of 62 M-M hips revised for suspected adverse reactions to metal debris. Based on the degree of organisation of lymphoid aggregates, it was possible to define three broad but distinct lymphocytic categories; diffuse infiltrates, T cell aggregates, and organised lymphoid-like aggregates.

Organised lymphoid-like aggregates have been termed tertiary lymphoid organs (TLOs) because leukocytes are organised within the foci into structures similar to those observed in secondary lymphoid organs (SLOs). Features associated with TLOs comprise centrally placed B cell aggregates with presence of a FDC network, loosely surrounded by T cells, local expression of lymphoid chemokines and cytokines as well as presence of plasma cells and PNAd+ HEVs. This morphological, cellular and molecular organisation is believed to be pathogenic in mounting a sustained immune response to persistent antigens in chronic inflammatory conditions [23].

Similar to TLOs found in other chronically inflamed tissues, the organised lymphoid-like aggregates in periprosthetic tissues of M-M implants showed some degree of T/B cell segregation with FDC networks placed in the B cell rich areas indicative of a germinal centre (GC)-like reaction. The lymphoid aggregates contained a surprisingly high percentage of B cells (40%). The presence and abundance of B cells did not correlate with the degree of segregation in T/B cell areas of the aggregates. Interestingly, FDC networks were also observed in non-segregated foci (however they were strictly associated with B cell rich areas), suggesting that FDC network formation is independent to T/B segregation in this pathology. This data is in contrast with previous reports on TLOs in other organs where FDC presence was detected selectively in segregated foci [28], [31].

Specialised stromal cells play an important role in chronic inflammation and disease persistence [41]. Studies in human and mouse models have shown that the stroma in ectopic TLOs is activated and provides the necessary signals for the molecular events that lead to organisation and maintenance of the infiltrating immune cells [42]. We detected expression of one such stromal cell marker, gp38 or podoplanin, in M-M tissues containing lymphoid aggregates [42], [43]. In tissues of M-M implants with organised lymphoid follicles, podoplanin expression was detected in lymphatic vessels and within the aggregates in a characteristic reticular pattern. Surprisingly, unlike SLOs and some other TLOs, we did not detect podoplanin expression on T-zone fibroblastic reticular cells (TRCs). Podoplanin staining was, however, detected in the B cell rich area and showed a reticular pattern of staining that co-localised with the FDC networks.

FDC networks within GCs play an important role in augmenting B cell immune responses by trapping and presenting the antigen and favouring B cell proliferation and differentiation into affinity-selected B cells and plasma cells [44]. It is tempting to postulate a similar role for FDC in this context. The origin of FDC in peripheral tissue is not known. Recently evidence that they might derive from PDGF-R+ pericytes has been provided [45]. Our data, highlighting the difference in podoplanin expression between SLOs and TLOs, suggest that there may be different origins of FDC in secondary and tertiary lymphoid structures.

Formation of tertiary lymphoid organs is classically associated with modification of vascular endothelium which acquires a plump morphology and upregulates the expression of adhesion molecules such as ICAM1, VCAM1, MADCAM1, PNAd. This modification aids the recruitment of circulating immune cells into the site of chronic inflammation [35], [46]. HEVs are best marked by expression of peripheral lymph node addressin (PNAd) and are usually detected within T cell areas and between the follicular regions of secondary lymphoid organs and TLOs [24], [28], [46]. This pattern of PNAd+ HEVs was also found in the periprosthetic tissues of M-M implants having large organised lymphoid-like aggregates, further reinforcing the notion that periprosthetic tissues of some ALVAL cases recapitulate features of TLOs.

We also investigated the expression of lymphoid homing chemokines, CCL21 and CXCL13, shown to be critical for T and B cell organisation and maintenance in SLOs and TLOs [24], [27], [28], [36]. Consistent with other reports [24], [27], [28], we found increased expression of CXCL13 within the B cell rich areas and in co-localisation with the FDC network. While the expression of CXCL13 has been shown to be a predictive factor for progressive organisation of lymphoid aggregates, its expression has also been detected in the absence of full lymphoid organisation indicating an upstream role for this molecule in TLO formation [25], [26], [28]. In contrast to CXCL13, the expression of CCL21 was only scarcely detected in the T cell areas of some large lymphoid like aggregates in periprosthetic tissues of M-M implants.

In several autoimmune diseases such as RA and Sjogren's syndrome, the formation of fully mature GC displaying FDCs networks and ectopic lymphoid chemokine expression has been associated with disease severity and local production of autoantibodies [26], [47], [48]. In M-M implants, metal ions and nano-sized particles are generated alongside microscopic wear debris. It is suggested that ions and nano-particles have the potential to form complexes with native proteins to generate an immunogenic hapten-carrier complex. Presentation of the hapten-carrier complex on locally differentiated FDCs, may lead to local breakdown of self-tolerance, generation of auto-reactive B cells and production of auto-antibodies. The observed significant increase in the regional concentration of Co and Cr with the T and B cell areas supports this concept. Recent ex-vivo analyses of metal ion speciation in periprosthetic tissues from failing M-M joints have suggested Co to be more likely to form peptide associations than Cr in the periprosthetic environment with the later more likely to form insoluble inorganic precipitates; however ALVAL cases were not systematically identified [49]. In this case, the increases in implant element concentrations in these regions cannot be accounted for by accumulation in professional phagocytes which have been extensively demonstrated to be associated with CoCr implant metal derivatives in synovial tissues [6], [9], [49]. Our findings also demonstrate an increase in relative concentration of Cr when compared with Co within TLO structures which is suggestive of its importance in ALVAL cases. In further support of this hypothesis, plasma cells were also found in the periprosthetic tissues with large organised lymphoid-like aggregates. Accordingly, Baff and April, key regulators of B cell homeostasis [39], were also found to be expressed within the aggregates, thus providing the functional machinery for local plasma cell differentiation and survival. Nonetheless, further studies are needed to address the origin and potential antibody repertoire of the plasma cells inhabiting the reactive tissue in M-M implants.

In conclusion, we have demonstrated for the first time that the lymphoid aggregates associated with ALVAL in a subset of patients having M-M hip implants display all the features associated with TLOs. While the role of T cells and macrophages has been well established in the reaction to M-M implants, the presence and importance of B cells has been largely underestimated in the cellular reaction to metal wear debris. In RA, it is known that B cells not only play a role in autoantibody production but are also required for macrophage and T cell activation and production of pro-inflammatory cytokines [50]. The significance of B cells and TLO formation in M-M implant pathology and its correlation with disease severity and outcome requires further evaluation. Similarly, studies to elucidate the association between tertiary lymphoid organ formation and autoimmunity to metal-hapten carrier complexes and/or T cell-mediated inflammatory responses are needed in order to develop novel therapies to overcome the clinical complications associated with M-M implants.
